# Face and Foot Deformities

**Published:** 1885-09

**Authors:** 


*Face and Foot Deformities.* By FREDERICK CHURCHILL. Pp. 195. London: J. & A. CHURCHILL. 1885.

Mr. Churchill tells us that he has associated the face with the foot in his monograph "chiefly because deformities of these members of the body being more manifest than deformities elsewhere, they constitute a greater hindrance to success in life." He justifies his right to teach on the subject in the following involved, not to say ungrammatical, sentence: "I may add that, as Surgeon to the Evelina Hospital for Children, these deformities of the body have occupied much of my thought and attention, with an earnest desire to erase from the chapter of accidents as many as possible of such unsightly disfigurements; so that in the inevitable struggle for supremacy, in after life, these poor children may stand a better chance of competing for the prizes, and not be so heavily handicapped in the race by their more fortunate competitors." Over the page we are told that "there are an endless variety of congenital deficiencies." Finding this sort of writing in the short preface, we naturally look for much more of it in the body of the book; and we are not disappointed. Mr. Churchill is by no means unique in the freedom with which he handles the English language. Indeed, want of the ordinary educational training of the cultured gentleman is daily becoming more painfully prominent in our profession. If our examining bodies were to demand a little more in the preliminary training, they might reasonably expect a little more in the professional pass; and then, instead of a cry for levelling down to meet the ordinary capacity of the uneducated student, we might have a demand for a levelling up to suit the culture of what has been called a learned profession.

This by the way, however.

The matter of the book is good. The descriptions of the deformities are clear and suggestive, and the treatment always sound, and occasionally novel. His plan of treating port-wine mark, by transfixing the larger vascular trunks with a needle cautery, seems to be promising. For the treatment of some of the minor deformities, especially such as are caused by skin diseases, many useful practical hints may be found. His advice in the treatment of club-foot has the genuine ring of practical common-sense. He says: "I must confess that I am a great enemy of the surgical appliance maker in the treatment of these cases—a business which, under the high-sounding title of orthopædic mechanician, is, I believe, very remunerative." We did not again select the sentence because it was ungrammatical, but because it expressed an opinion which we most cordially endorse. The value of manipulation and encasement in plaster of Paris is fully recognised, and sufficiently explained.

The drawings, some of them full-page lithographs, are decidedly good, and the general get-up of the work—as to paper, printing, and binding—is faultless.

